# Emerging Issues in Infective Endocarditis

**DOI:** 10.3201/eid1006.030848

**Published:** 2004-06

**Authors:** Beverley C. Millar, John E. Moore

**Affiliations:** * Belfast City Hospital, Belfast, Northern Ireland, United Kingdom

**Keywords:** endocarditis, diagnosis, PCR, 16S rRNA, historical, fungi, bacteria, molecular, valve

## Abstract

Infective endocarditis, a serious infection of the endocardium of the heart, particularly the heart valves, is associated with a high degree of illness and death. It generally occurs in patients with altered and abnormal heart architecture, in combination with exposure to bacteria through trauma and other potentially high-risk activities involving transient bacteremia. Knowledge about the origins of endocarditis stems from the work of Fernel in the early 1500s, and yet this infection still presents physicians with major diagnostic and management dilemmas. Endocarditis is caused by a variety of bacteria and fungi, as well as emerging infectious agents, including *Tropheryma whiplei*, *Bartonella* spp., and *Rickettsia* spp. We review the evolution of endocarditis and compare its progression with discoveries in microbiology, science, and medicine.

Endocarditis is a noncontagious chronic infection of the valves or lining of the heart, mainly caused by bacteria, although fungi can also be associated with this infection (1). The risk of infection of heart valves in persons predisposed to acquiring infective endocarditis increases with the following conditions: congenital heart disease, rheumatic fever, major dental treatment, open heart surgery, and genitourinary procedures. New evidence is growing that changes in social behavior, such as an increase in the incidence of body piercing, excessive alcohol consumption, and the use of intravenous self-administered illicit drugs may also predispose a susceptible person to an increased risk of acquiring endocarditis. The patient may exhibit any of the following signs and symptoms: fatigue and weakness; weight loss; fever and chills; night sweats; heart murmur; aches and pains; painful nodes in the pads of fingers and toes; red spots on skin of palms and soles; nail abnormalities; swelling of feet, legs, and abdomen; shortness of breath with activity; and blood in the urine. A medical history, physical examination, and echocardiogram are usually performed. Blood samples are usually taken, and the physical and biochemical properties of the blood are investigated. Endocarditis is usually curable provided an early diagnosis is made, and the patient receives the appropriate antimicrobial treatment; the time needed for recovery is approximately 6–8 weeks. The patient generally requires long-term antimicrobial drugs (4–6 weeks), hospitalization, and in some cases, valve replacement. A number of complications may be associated with the disease such as blood clots, stroke, heart rhythm problems, abscesses, and other infections. Infective endocarditis is associated with severe illness and death and generally occurs in patients with altered and abnormal heart architecture who have been exposed to bacteria through trauma and other potentially high-risk activities.

In 1885, Sir William Osler presented three Gulstonian Lectures on the topic of malignant endocarditis, which gave a comprehensive account of the disease and outlined the difficulties in its diagnosis (2). The disease had, in fact, been described by a French Renaissance physician, Jean François Fernel, approximately 350 years previously (3). More than 100 years after Osler's lectures, this serious infection can still remain a diagnostic and therapeutic dilemma. Its name has been changed several times, first to "bacterial endocarditis" and subsequently to "infective endocarditis" after the observation that microbiologic agents other than bacteria may cause the disease. In the early years of the new millennium, infective endocarditis still proves to be difficult to diagnose and is associated with a high death rate (21%–35%). Although many developments have taken place with respect to antimicrobial drug therapy in the treatment of the disease, its incidence is continuing to rise, with 3.3 cases per 100,000 population per year in the United Kingdom, with similar figures for the United States and 1.4–4.0 cases per 100,000 population per year in Europe as a whole (4). The reasons for this rise are the following: 1) longer survival of patients with degenerative heart diseases, 2) increased use of antibiotics, 3) increased incidence of prosthetic heart valves, 4) congenital heart disease in younger children, 5) increase in bicuspid valve disease, 6) advances in medical and surgical treatments, 7) increase in the number of injection drug users, and 8) more sensitive and specific diagnosis. Generally, the incidence is higher in men than in women (2:1), and the average age group affected is in the fifth decade (2).

## Historical Perspective

A historical description of developments in endocarditis closely reflects concurrent developments in laboratory medicine, particularly microbiology. Much of the innovations and developments relating to infective endocarditis were made by physicians in Europe, particularly in France (Table A1). Important contributions were, however, made by several German physicians, particularly in association with the birth of bacteriology (Table A1). More recently, the United States has played a strong role in helping define guidelines and diagnostic criteria that facilitate diagnosing infective endocarditis, including the Beth Israel (5), Duke (6) (Table 1), and modified Duke criteria (7,8) (Table 2). In addition, the American Heart Association has published several seminal articles on the antibiotic treatment and prevention of infective endocarditis (9).

**Table 1:** Original Duke criteria for the diagnosis and classification of infective endocarditis^a^

Major criteria	Minor criteria	Diagnosis
1. **Positive blood culture** i) Typical organism in >2 blood cultures in the absence of a primary focus (*Staphylococcus aureus*, enterococci, viridans streptococci, *Streptococcus bovis*, HACEK) ii) Persistently positive blood culture drawn more than 12 h apart or all ¾ drawn at least 1 h apart between first and last	1. **Predisposition** Heart condition Drug abuse	1. **Definite** 2 Major 1 Major and 3 minor 5 Minor pathologic/histologic findings
2. **Evidence of endocardial involvement** i) Positive echocardiogram (TOE) Oscillating intracardiac mass on valve, implanted material or supporting structures in path of regurgitant jets Abscess New partial dehiscence of prosthetic valve ii) New valvular regurgitation	2. **Fever** >38°C	2. **Possible** Findings fell short of the definite but not rejected categories
	3. **Vascular phenomena** Major arterial emboli Janeway lesions Septic pulmonary infarcts	3. **Rejected** Alternate diagnosis Resolution of the infection with antibiotic therapy for <4 days No pathologic evidence after antibiotic therapy
	4. **Immunologic phenomena** Osler's nodes Roth spots Rheumatoid factor Glomerulonephritis	
	5. **Microbiologic evidence** Positive blood culture not meeting major criteria Positive serologic finding	
	6. **Endocardiographic evidence** Consistent with infective endocarditis but not meeting the major criteria	

^a^Source (6); HACEK, *Haemophilus aphrophilus*, *Actinobacillus actinomycetemcomitans*, *Cardiobacterium hominis, Eikenella corrodens*, and *Kingella kingae* group; TOE, transesophageal echocardiogram.

**Table 2:** Recent suggested modifications to the Duke criteria for the diagnosis of infective endocarditis (IE)^a^

Microbiologic	Biochemical	Clinical
*Blood culture* Bacteremia due to *Staphylococcus* *aureus* should be considered a major criterion regardless of whether the infection is nosocomially acquired or whether a removable source of infection is present *Serology* Positive for *Coxiella burnetii* (major criterion) Positive for *Bartonella* spp. Positive for *Chlamydia* spp. *Molecular* Evidence for the presence of bacterial or fungal DNA in blood or valve material (major criterion)	Elevated level of CRP >100 mg/L Elevated ESR defined as more than one and a half times higher than normal, i.e., >30 mm/h for patients <60 years of age >50 mm/h for patients >60 years of age	Possible endocarditis now defined as one major and one minor criterion or three minor criteria Omission of criterion "echocardiogram consistent with IE but not meeting major criterion" Newly diagnosed clubbing Evidence of splinter hemorrhages Petechiae Microscopic hematuria (disregarded for patients with positive urine cultures, menstruating women, patients with end- stage renal disease and patients with urinary catheters) Presence of central nonfeeding venous lines or peripheral venous lines (minor) Purpura

^a^Sources (7,8); CRP, C-reactive protein; ESR, erythrocyte sedimentation rate.

For approximately the first 200 years after the disease was initially described, the anatomy of the heart and heart valves in the diseased state of infective endocarditis was comprehensively elucidated in medical anatomical sketches made after postmortem examination. (For a comprehensive account of the early description of endocarditis, see Contrepois [10].) Not until the early to mid-1800s were descriptions recorded of the medical signs and symptoms of the disease in live patients. Such descriptions included the detection of cardiac murmurs, after percussion and auscultation. Detection of such murmurs was aided by the development of the stethoscope in 1816. From 1830 to 1840, elevated body temperature was recorded as an important symptom of the disease. However, not until the late 1800s and early 1900s was a comprehensive synthesis of information formed by various scholars in Europe and North America, including Sir William Osler in Canada (2) and Thomas Horder in England (11) (Table A1). Osler and Horder were instrumental in establishing fundamental mechanisms regarding the pathophysiology of infective endocarditis and are, to a large degree, responsible for how we view endocarditis today. The Figure and Table A1 chronologically map the history of infective endocarditis, including diagnostic developments, treatment, and prevention, and emerging causal agents.

**Figure:**
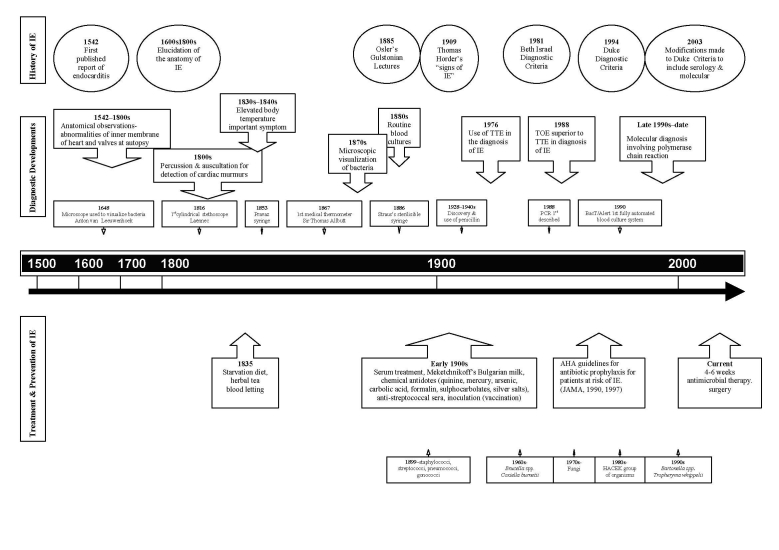
Historial timeline describing concurrent developments regarding the history of emerging causal agents of infective endocarditis (IE), diagnostic developments, treatment options, and diversity of causal agents. Download PDF.

The birth of bacteriology as a separate discipline of pathology gave rise to the introduction of the important description of microbiology in the etiology of infective endocarditis. With the early technical innovations of Pasteur in France in the 1880s, routine blood cultures were introduced in the late 19th century as an important part of laboratory investigation into the microbiologic causes of infective endocarditis. Although causal agents of infective endocarditis could now be detected and clearly described, little could be achieved in terms of their eradication because the existence of antibiotics was as yet unknown. However, in Germany, Gerard Domagk, bacteriologist and pathologist, was appointed as director of the I.G. Farbenindustrie (Bayer) Laboratory for Experimental Pathology and Bacteriology in Wuppertal in 1925. Domagk was innovative in that he began to experiment with dyes, looking for their possible effects against various infections. He described the effect of prontosil red against streptococcal infections in mice; the active component of prontosil was later described as sulfanilamide. At approximately the same time, Sir Alexander Fleming discovered the antibacterial effects of a secondary metabolite (penicillin), produced from a filamentous fungus. Such discoveries were revolutionary because medicine now had an effective means of treating bacterial infections, including infective endocarditis, caused by a wide variety of bacterial pathogens, most notably *Streptococcus* species. Since wild-type pathogens had not had sufficient time to develop resistance to these newly described antimicrobial agents, treatment failures due to resistance were infrequent. Fleming did observe, however, that some organisms were resistant to penicillin and suggested that the phenomenon be followed up. Approximately 60 years later, the marked increase in resistance to antimicrobial agents is cause for concern on all continents. The tangible consequence is that clinicians may have fewer antimicrobial agents to treat both benign and serious infections, including infective endocarditis. To combat the threat of such a "postantibiotic era," the global pharmaceutical industry has responded by producing novel antimicrobial agents, including the carbapenems (imipenem and ertapenem), the oxozolidones (linezolid), and improved antifungal agents (caspofungin and voriconazole), which prolong antimicrobial effectiveness before the problem of resistance evolves with such new agents.

Over the past century, streptococci and staphylococci have remained the main causative organisms associated with infective endocarditis, with an increase in cases due to staphylococci associated with injection drug users and HIV patients. With substantial advances made in the isolation and identification of microorganisms, scientists now recognize a wide spectrum of causal organisms. Although rare, infective endocarditis is caused by gram-negative organisms such as the HACEK (*Haemophilus aphrophilus*, *Actinobacillus actinomycetemcomitans*, *Cardiobacterium hominis*, *Eikenella corrodens*, *Kingella kingae*) group, *Bartonella* spp., and *Coxiella burnetii*. More recently, cases of fungal endocarditis have increased, particularly in postoperative patients, injection drug users, and immunocompromised patients (4).

A history of rheumatic fever can serve as a risk factor for acquiring infective endocarditis. The incidence of rheumatic fever, which was common as recently as a century ago, is relatively rare today (12). This decline in the incidence of rheumatic fever has not been mirrored by a pro rata decrease in the incidence of infective endocarditis, which suggests that additional etiologic factors are becoming more important in acquiring endocarditis.

## Current Trends and Future Concerns

Although endocarditis has been documented for approximately 450 years, the diagnostic challenges and treatment dilemmas are as real today as they were in the time of Fernel (3). Major advances have been made in the diagnosis of endocarditis, in both laboratory and clinical (imaging) parameters, but we are witnessing the emergence of several newly described causal bacterial species, such as *Tropheryma whipplei* and *Bartonella* spp., as well as sporadic case reports of unusual and uncommon causal organisms, including *Finegoldia* sp., *Gemella* spp., and *Abiotrophia defectiva*. In addition, since diagnostic methods, mainly 16S rDNA polymerase chain reaction (PCR) and sequencing, are now beginning to identify such infections, no evidence base exists to help determine effective antimicrobial drug regimens to successfully treat endocarditis caused by such organisms. Furthermore, as specimens from many of these infections are culture-negative, conventional antibiotic susceptibility testing does not help the cardiologist decide on the most suitable antimicrobial drug regimens. Another current concern is that we may be returning to a time in which we are largely unable to successfully treat simple infections from panresistant organisms, a scenario that some have described as the postantibiotic era. Indeed, in Northern Ireland, we have now witnessed our first cases of penicillin-resistant pneumococcal meningitis and endocarditis. The increasing incidence of congenital heart disease in children and changing social trends accentuate risk factors for endocarditis.

Endothelial cell dysfunction, resulting from a combination of atypical mechanical forces due to altered cardiac architecture and microbial infection, may lead to an episode of infective endocarditis. Because the endothelium helps regulate vascular tone, inflammation, thrombosis, and vascular remodeling, any insult to the host endothelium may result in infective endocarditis, in which the valves may show changes in the synthetic, morphologic, and metabolic functions of the valvular endothelial cells (13).

## Cases in Well-known Persons

Although a relatively uncommon infection, infective endocarditis has been the primary cause of death of several well-known persons, particularly those involved with the arts. One of the late 19th and early 20th century's most influential composers, Gustav Mahler (1860–1911), died from streptococcal endocarditis (10,14). The first sign of valvular problems was observed in 1907, where a compensated mitral contraction was noted. For the next 3 years, he showed little evidence of symptoms of valvular disease until late 1910, when he spent Christmas and the New Year's holiday nursing a sore throat. He was in New York City where he conducted a Philharmonic Orchestra concert on January 17, including the first performance of a revised version of his fourth symphony. On February 24, he became ill with endocarditis, initially diagnosed as influenza. He was attended by one of the most prominent physicians in the city, Emanuel Libman, an important exponent of the value of bacterial blood cultures. Libman demonstrated the presence of viridans streptococci in a large volume (200 mL) of blood drawn from Mahler. Mahler's initial treatment consisted of a "serum treatment" of the times, as well as Metchnikoff's Bulgarian Milk. The latter treatment appeared to work, until early May when blood cultures returned positive with viridans streptococci. The endocarditis was now very marked, with septic abscesses beginning to appear in other parts of his body. On May 18, Mahler died. His untimely death prevented society from hearing him conduct a completed version of his tenth symphony as well as his own opportunity to hear the first public performance of his ninth symphony, which took place on June 26, 1912, by the Vienna Philharmonic Orchestra.

Ottorino Respighi (1879–1936) was an Italian composer who died at the age of 57 from endocarditis. The first signs of Respighi's endocarditis were noted in late 1935, when he was working on his opera Lucrezia; at that time, he was observed to be extremely fatigued, but the cause was unknown (14). In January 1936, *S. viridans* endocarditis was noted when this organism was isolated from his blood. Although sulfonamide drugs were dispatched from Berlin for his treatment, the treatment was unsuccessful, possibly due to the advanced stages of sepsis.

One of Scotland's most famous poets, Roberts Burns (1759–1796), perhaps best known for writing Auld Lang Syne, also had infective endocarditis. He died in July 1796 at the age of 37 years (15). Some historians claim that Burns's work in his teenage years on his father's tenant farm in southwest Scotland did the primary damage to his health. However, Burns's history of rheumatic fever likely predisposed him to infective endocarditis. Burns was attended medically by William Maxwell (1760–1834), who described Burns's symptoms as "flying gout" and prescribed sea-bathing in country quarters and horse riding, so-called cures that probably hastened Burns's death. However, Burns's affinity for alcohol may have contributed to the suppression of his immune system, thus hastening the illness and ultimately his death.

One of the most famous physicians to die of endocarditis was Alois Alzheimer (1864–1915). Alzheimer is most widely known for his description of an "unusual disease of the cerebral cortex," which affected a woman in her fifties, causing memory loss, disorientation, hallucinations, and ultimately her death at age 55. The disease was named after him by his senior mentor at the Munich Medical School, Emil Kraepelin. Alzheimer was also cofounder and copublisher of the journal Zeitschrift für die gesamte Neurologie und Psychiatrie. Alzheimer's last position was professor of psychiatry at the University of Breslau (now Wroclaw, Poland), which he held for the last 3 years of his life. Historians report that a severe cold was the beginning of Alzheimer's final illness, but endocarditis was responsible for his death at the age of 51 years (16).

Orville Gibson, guitar manufacturer (1856–1918), was another musician who died from endocarditis (17). Gibson's patent contained his ideas for the construction of a mandolin with a carved top and back and with sides, which were constructed from a solid section of wood rather than from thin strips. In 1902, Gibson's physical and mental health began to fail, and he had a history of poor health until 1911. He returned to the St. Lawrence State Hospital, Ogdensburg, New York, in August 1916, a psychiatric center. On August 21, 1918, Gibson died of endocarditis while a patient in the institution.

Rudolph Valentino (1895–1926), a famous actor of the silent screen, also had endocarditis, which also led to his death (18). Valentino had a perforated gastric ulcer closed on August 15, 1926; however, he died from endocarditis on August 23, 1926, at the age of 31 years.

More recently, endocarditis has been described as the cause of death for John Glascock (1951–1997), the recording bass player with the rock band Jethro Tull. Glascock had a tooth abscess, which was believed to be the site of entry for an infectious agent that caused endocarditis. Endocarditis developed in Brian Littrell (1975– ), singer with the Backstreet Boys, at the age of 5 years (he was born with a ventricular septal defect, although surgery was not recommended at the time) (19). Brian was admitted to St. Joseph's Hospital, Lexington, Kentucky, where he received extensive intravenous therapy. Endocarditis also developed in a young American actor, Sebastian Hitzig, after he accidentally stepped on a toothpick contaminated with *Staphylococcus aureus*.

In conclusion, considering infective endocarditis to be an "emerging" problem in the 21st century may seem unusual, given that the illness has been well documented over the last 450 years. However, such emergence can be attributed to several factors: 1) the emergence of antimicrobial resistance in classic infective endocarditis microflora, namely, the gram-positive cocci; 2) the existence of antimicrobial resistance in complex ecologic biofilms; 3) the changing pattern of causal agents now regarded as important pathogens of infective endocarditis, e.g., *Bartonella* spp., *T. whipplei*, and fungi; and 4) changing epidemiologic trends of persons who acquire infective endocarditis, including injection drug users, persons with HIV/AIDS, children with congenital heart defects, and persons undergoing body piercing. Furthermore, the way we provide inpatient medical care has also been associated with the emergence of nosocomial infective endocarditis, which can result from invasive procedures such as catheterization, although no cardiac surgery has been performed. The next 100 years will likely witness the emergence of even more changing trends of infective endocarditis, which as yet have not been well recognized.

Although this "old" disease has evolved over the last 450 years, diagnostic and treatment options have developed in tandem, and the prognosis of this disease has markedly improved. However, the emergence of novel etiologic agents, changing social trends, and increased antimicrobial resistance have allowed this disease to remain evasive, which will require new approaches, particularly relating to treatment options in the future.

## Supplementary Material

Print Ready Figure Historial timeline describing concurrent developments regarding the history of emerging causal agents of infective endocarditis (IE), diagnostic developments, treatment options, and diversity of causal agents.

## Appendix

**Table A1:** Chronology of important scientific and medical events in the history of infective endocarditis

Year	Scientist/physician, Country	Major findings
1554	Jean François Fernel, France	Earliest report of endocarditis in book Medicini
1669	Richard Lower, England	Accurately described tricuspid valve endocarditis
1646	Lazarus Riverius, France	Described unusual "outgrowths" from autopsy of patient with endocarditis; detected murmurs by placing hand on patient's chest
1708	Giovanni Maria Lancisi, Italy	Described unusual structures in entrance of aorta
1715	Raymond Vieussens, France	Described abnormality in aortic mitral valve
1749	Jean-Baptiste Sénac, France	Described valvular lesions
1769	Giovanni Battistu Morgagni, Italy	Linked infectious disease and endocarditis; observed association with the spleen
1784	Eduard Sandifort, France	Accurately drew intracardiac abnormalities
1797	Matthew Baillie, England	Showed relationship between rheumatism and heart disease
1799	Xavier Bichat, France	Described inflammatory process associated with endocarditis
1806	Jean Nicholas Corvisart, France	Described unusual structures in heart as "vegetations," syphilitic virus as causative agent of endocarditis, and theory of antiviral treatment of endocarditis
1809	Allan Burns, England	Indicated vegetations were not "outgrowths" or "buds" but particles adhering to heart wall
1815	Friedrich Kreysig, Germany	Elucidated inflammatory processes associated with endocarditis
1816	Théophile Laënnec, France	Invented cylindrical stethoscope to listen to heart murmurs; dismissed link between venereal disease and endocarditis
1832	James Hope, England	Confirmed Laënnec's observations
1835–40	Jean-Baptiste Bouillaud, France	Named endocardium and endocarditis; described symptoms; prescribed herbal tea and bloodletting as treatment regimen; described link between acute rheumatoid arthritis and endocarditis
1852	William Senhouse Kirkes, England	Described consequences of embolization of vegetations throughout body. Described cutaneous nodules (named "Osler's nodes" by Libman)
1858–71	Rudolph Virchow, Germany	Examined fibrin vegetation associated with endocarditis by microscope; coined term "embolism;" discussed role of bacteria, vibrios, and micrococci in endocarditis
1861	Jean-Martin Charot, France	Confirmed Virchow's theory on emboli
1861	Alfred Vulpian, Germany	Confirmed Virchow's theory on emboli
1862	Etienne Lancereaux, France	Described granulations or foreign elements in blood and valves, which were motile and resistant to alkalis
1868–70	Samuel Wilks, England	Described infected arterial blood as originating from heart; proposed scarlet fever as cause of endocarditis
1869	Emmanuel Winge, Norway	Established "parasites" on skin transported to heart and attached to endocardium; named "mycosis endocardii"
1872	Hjalmar Heiberg, Norway	Detected microorganisms in vegetations of endocarditis
1878	Edwin Klebs, Germany	All cases of endocarditis were infectious in origin
1878	Ottomar Rosenbach, Germany/Poland	Combined experimental physiology and infection to produce animal model of endocarditis in rabbit; noted valve had to be damaged before bacteria grafted onto valve
1878	Karl Koester, Germany	Micrococci enter vessels that valves were fitted into; valves exposed to abnormal mechanical attacks over long period created favorable niche for bacterial colonization
1879	Joseph Hamburg, Germany	Virchow's student; employed early animal model of endocarditis
1879	Germain Sée, France	Proposed etiology of endocarditis was based on infectious model and treatment should focus on eliminating "parasitic infection"
1880	Jacques Doleris, France	Working with Pasteur, proposed use of routine blood cultures
1881–86	Arnold Netter, France	Believed endocarditis could appear during various infections; noted translocation of respiratory pathogen from pulmonary lesion to valve through blood
1883	Michel Peter, France	Believed microorganisms were result, not cause, of endocarditis
1884	Joseph Grancher, France	Named disease "infective endocarditis"
1886	Valimir Wyssokowitsch and Johannes Orth, Germany	Demonstrated various bacteria introduced to bloodstream could cause endocarditis on valve that had previous lesion
1885	Sir William Osler, Canada	Synthesized work of others relating to endocarditis
1899	Hermann Lenhartz, Austria	Described streptococcal, staphylococcal, pneumococcal, and gonococcal endocarditis
1903	Hugo Schottmüller, Germany	First described "endocarditis lenta"
1909	John Alexander Mullen, Canada	Credited by Osler as first to observe cutaneous nodes (named "Osler's nodes" by Libman) in patients with endocarditis
1909	Sir Thomas Horder, England	Analyzed 150 cases of endocarditis and published diagnostic criteria relating to signs and symptoms
1910	Emmanual Libman, USA	Described initial classification scheme to include "subacute endocarditis," with clinical signs/symptoms; absolute diagnosis required blood cultures
1981	Von Reyn, USA	Described Beth Israel criteria based on strict case definitions
1994	David Durack, USA	New criteria utilizing specific echocardiographic findings
1995	American Heart Association, USA	Antibiotic treatment of adults with infective endocarditis caused by streptococci, enterococci, staphylococci, and HACEK^a^ microorganisms
1996	Pierre Fournier, France	Modified Duke criteria to allow serologic diagnosis of *Coxiella burnetii*
1997	American Heart Association, USA	Guidelines for preventing bacterial endocarditis
1997	Lamas and Eykyn, UK	Suggested modifications to Duke criteria for clinical diagnosis of native valve and prosthetic valve endocarditis: analysis of 118 pathologically proven cases
1998	Working Party of the British Society for Antimicrobial Chemotherapy, UK	Guidelines for antibiotic treatment of streptococcal, enterococcal, and staphylococcal endocarditis
1998	Endocarditis Working Group of the International Society for Chemotherapy, Europe	Antibiotic treatment of infective endocarditis due to viridans streptococci, enterococci, and other streptococci; recommendations for surgical treatment of endocarditis
2000	Jennifer Li, USA	Updated and modified Duke criteria
2002	Beverley C. Millar, UK	Modified Duke criteria to include a molecular diagnosis of causal agents (20)
2001–2003	Didier Raoult, France	Described etiology of *Bartonella* spp., *Tropheryma whipplei*, and *Coxiella burnetii* in endocarditis

^a^HACEK, *Haemophilus aphrophilus*, *Actinobacillus actinomycetemcomitans*, *Cardiobacterium hominis*, *Eikenella corrodens*, *Kingella kingae* group, *Bartonella* spp., and *Coxiella burnetii*.
