# Supplementary figures and images for: Emerging Issues in Infective Endocarditis
Source: Emerg Infect Dis. 2004 Jun;10(6):1110–6. doi: 10.3201/eid1006.030848 (PMC3323180; doi:10.3201/eid1006.030848)

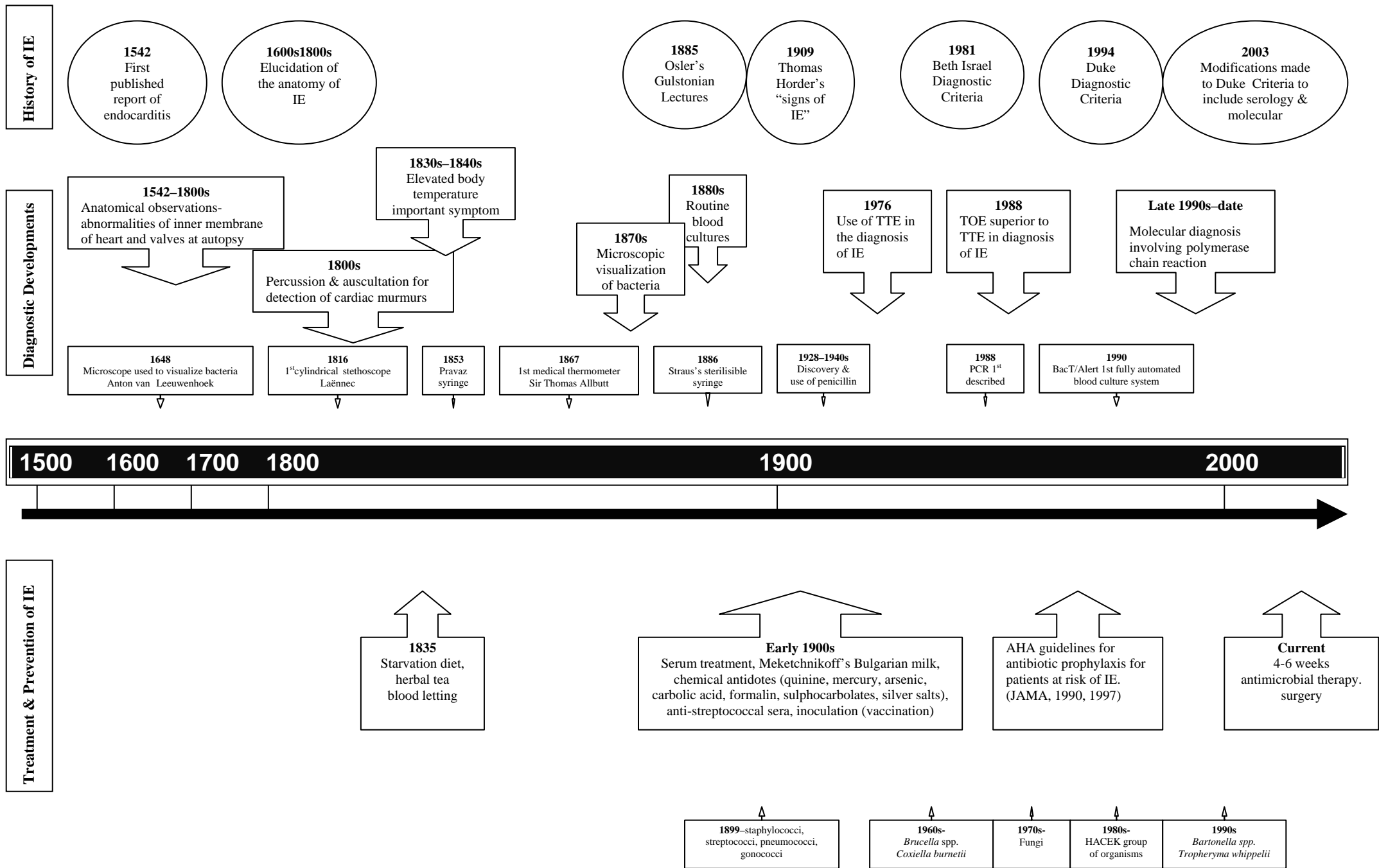

Supplement: Print Ready Figure — Historial timeline describing concurrent developments regarding the history of emerging causal agents of infective endocarditis (IE), diagnostic developments, treatment options, and diversity of causal agents. [file 03-0848-F.pdf]
